# The ego in psychedelic drug action – ego defenses, ego boundaries, and the therapeutic role of regression

**DOI:** 10.3389/fnins.2023.1232459

**Published:** 2023-10-06

**Authors:** Tobias Buchborn, Hannes S. Kettner, Laura Kärtner, Marcus W. Meinhardt

**Affiliations:** ^1^Institute of Psychopharmacology, Central Institute of Mental Health, Medical Faculty Mannheim, University of Heidelberg, Heidelberg, Germany; ^2^Centre for Psychedelic Research, Department of Medicine, Imperial College London, London, United Kingdom; ^3^Psychedelics Division, Neuroscape, Department of Neurology, University of California, San Francisco, San Francisco, CA, United States; ^4^Department of Molecular Neuroimaging, Central Institute of Mental Health, Medical Faculty Mannheim, University of Heidelberg, Heidelberg, Germany

**Keywords:** psychedelics, ego regression, ego defenses, ego boundary, character, psycholytic therapy, psychedelic-assisted psychotherapy, depth psychology

## Abstract

The ego is one of the most central psychological constructs in psychedelic research and a key factor in psychotherapy, including psychedelic-assisted forms of psychotherapy. Despite its centrality, the ego-construct remains ambiguous in the psychedelic literature. Therefore, we here review the theoretical background of the ego-construct with focus on its psychodynamic conceptualization. We discuss major functions of the ego including ego boundaries, defenses, and synthesis, and evaluate the role of the ego in psychedelic drug action. According to the psycholytic paradigm, psychedelics are capable of inducing regressed states of the ego that are less protected by the ego’s usual defensive apparatus. In such states, core early life conflicts may emerge that have led to maladaptive ego patterns. We use the psychodynamic term *character* in this paper as a potential site of change and rearrangement; character being the chronic and habitual patterns the ego utilizes to adapt to the everyday challenges of life, including a preferred set of defenses. We argue that in order for psychedelic-assisted therapy to successfully induce lasting changes to the ego’s habitual patterns, it must psycholytically permeate the characterological core of the habits. The primary working principle of psycholytic therapy therefore is not the state of transient ego regression alone, but rather the regressively favored emotional integration of those early life events that have shaped the foundation, development, and/or rigidification of a person’s character – including his or her defense apparatus. Aiming for increased flexibility of habitual ego patterns, the psycholytic approach is generally compatible with other forms of psychedelic-assisted therapy, such as third wave cognitive behavioral approaches.

## 1. Introduction

Psychedelic drugs such as psilocybin and LSD have, in recent decades, been object of renewed interest in the treatment of psychological disorders and it seems to be a widely accepted notion that the eponymous *mind-revealing* properties of the psychedelic experience [psychedelic, Gr. *psȳchḗ* mind, and *dēloũn* to reveal (Kraif, 2007)] revolve along alterations of the so-called *ego* (Carhart-Harris and Friston, 2010; Preller and Vollenweider, 2016). The phenomenon of *ego dissolution*, for instance, is an integral part of psychedelic peak experiences (Fox et al., 2018; Richards, 2021) and a key component in certain forms of psychedelic-assisted psychotherapy (Majić et al., 2015; Fuentes et al., 2020; Ko et al., 2022). Grof (1980b), a pioneering LSD therapist, viewed ego death and the transcendent “loss of boundaries between the subject and the objective world” as “the main objective of psychedelic therapy” (p. 35) (Grof, 1980b). Efforts to understand the neuronal substrates of ego dissolution (Carhart-Harris et al., 2016; Tagliazucchi et al., 2016; Stoliker et al., 2022), along with philosophical and phenomenological analyses (Letheby and Gerrans, 2017; Milliere, 2017; Deane, 2021), have been ongoing. Despite the suggested importance of ego dissolution in psychedelic drug action (Lebedev et al., 2015; Uthaug et al., 2018; Kałużna et al., 2022), however, the original psychodynamic formulation of the ego has received only little attention in the psychedelic research community (for exceptions: Carhart-Harris and Friston, 2010; Kraehenmann et al., 2017; Rabeyron, 2021; Fischman, 2022; Guss, 2022).

The delineation of the ego-construct from psychologically overlapping yet distinct constructs is often difficult, reflected in varying definitions like “sense of self,” “[…] immutable identity or personality” (p. 2) (Carhart-Harris et al., 2014) or “content of the phenomenal self-model” (p. 4) (Metzinger, 2009). Additionally, most psychometric measures used in the psychedelic research community focus on the phenomenology (i.e., aspects of subjective experience related to ego functioning) rather than the functionality of the ego *per se* (Scharfetter, 2003; Nour et al., 2016; Milliere, 2017; De Deus Pontual et al., 2022). The ego-construct has also been overshadowed by the prevalence of the *self* as an alternative construct in the current scientific discourse (e.g., Sass and Parnas, 2003; Cermolacce et al., 2007; Heatherton, 2011). The relative absence of the psychodynamic ego may be due to the general decrease in scientific impact of psychodynamic theories and the difficulty of their experimental validation compared to cognitive behavioral theories (Carhart-Harris et al., 2014). Consequently, the validation and discussion of the ego across disciplines remain ambiguous (Northoff, 2023b). However, first evidence has been presented that the psychodynamic ego can inform computational and neurophysiological data on psychedelic-brain interaction (Carhart-Harris and Friston, 2010, 2019).

This review aims to outline important psychological and psychiatric concepts related to the ego-construct, particularly in the context of psychodynamic ego and drive psychology.^1^ We discuss key functions of the ego frequently mentioned in the psychedelic literature (including ego boundaries, ego defenses, or the mental representation of the own person), explore psychodynamic models of psychedelic drug action, and examine initial findings on the underlying neurobiology. We propose that a disintegration of ego functions –with a loosening of ego defenses and a regression to functional modes characteristic of earlier phases of psychic development– is one of the major processes underlying the psychedelic experience. Specifically, we suggest that the psychedelic state reflects a temporary liberation of the ego from the constraints of its habitual mode of functioning, determined by the *character* of a person. Character, as it is used here, describes a psychological backbone structure that represents the routines through which the ego operates, including its preferred set of defenses. Building on the psycholytic approach to psychedelic-assisted therapy (Passie, 1997b; Passie et al., 2022), we hypothesize that it is *character* and its maturation that need to be targeted within a well-established and sustainable client-therapist alliance if a temporary ego regression as acutely induced by psychedelics is supposed to turn into sustained mental health improvements. With *character* conceptualized as reflecting the routines of the ego, and the mental representation of the own person conceptualized as a major downstream function of the ego, psychedelic-induced ego regression might be particularly well-suited for assisting psychotherapeutic treatments of disorders involving maladaptive routines, habits, compulsions, and/or chronic distortions of self-representation (e.g., personality disorders, addiction, obsessive-compulsive or eating disorders, depression, or phantom limb pain).

## 2. Psychodynamic conceptualization of the ego

The ego, as discussed in this review, is a psychodynamic concept rooted in Sigmund Freud’s structural model of the mind (1923) and that has been further developed by *ego psychologists* such as Freud (1942), Hartmann (1964), and Erikson (1987, 1998). In modern terms, the ego can be defined as a self-organizing, multifunctional process (Lettieri, 2005; Schamess and Shilkret, 2008) responsible for internal homeostasis, external adaptation (Auchincloss and Samberg, 2012), and the formation of mental representations that create a coherent sense of self (Kernberg, 1982). In this section we provide the theoretical background of the psychodynamic ego concept and prime for a subsequent evaluation of the psychedelic experience and its therapeutic implications from an ego-theoretical and psycholytic standpoint, respectively. We differentiate the ego from other psychological constructs (such as *self* and *character*) and discuss its major functions –including the secondary process, ego boundary, ego defenses, and the ego’s synthetic core function. Moreover, we explore functional ego alterations as they occur in regression and psychosis.

### 2.1. Drive psychology

Sigmund Freud, the founder of *psychoanalysis* and *drive psychology*, introduced the ego in his work “The Ego and the Id” (Freud, 1923/1961). According to Freud’s structural model, the psyche is governed by three agencies which during early development successively differentiate one by the other: The id, the ego, and the superego (Quinodoz, 2005). At first, the psyche is all *id* –an imminent *emergence* of the body that represents innate impulses and somatic needs. The ego, Freud theorizes, is born from the id in friction with the environment and *ab ovo* constituted as an interface to the external world: The “ego is first and foremost […] the projection of a surface” (p. 31) (Freud, 1923/1961). The superego evolves from the ego due to internalization of parental reprimands and serves as an authoritative agency, which in manifestation of the conscience causes friction to the ego. It is conceptually rooted in the acoustic sphere with once internalized word-representations of the parents later re-driven by id forces (Freud, 1923/1961, 1940/1964). The superego monitors the ego, compares its actions to the ego ideal –an internalized set of standards and *images of perfection*– and modulates ego functioning via mobilization of guilt or shame (Auchincloss and Samberg, 2012). The Freudian ego serves three masters –the instincts, the moral and social norms as internalized by the superego, and the demands of reality and the external world. A well-functioning ego mitigates conflicts between these forces and expands its organization (Freud, 1933/1964). The ego largely acts in the conscious realm, draws readily available mnestic elements from the preconscious realm into consciousness, and additionally exerts unconscious functions, for instance when applying *mechanisms of defense*. These will later be discussed in the context of *ego psychology*.

#### 2.1.1. Primary and secondary process

In Freudian theory, the functions of the ego are referred to as *secondary process*, the dynamics of the id as *primary process*. The primary process involves a free flow of mental energy and strives for immediate discharge (Holt, 2009). It is an information-processing system that primarily deals with affect-related excitation (Marcus, 1999). It is governed by unconscious wishes, urges, fantasies, and conflicts, allows for contradictions, and disregards logic and the reality of time (Auchincloss and Samberg, 2012). In contrast, the ego-governed secondary process represents an inhibitory mode of processing; it binds energy through mental representations, especially through thought and language, and thereby transforms it to “a higher dynamic level” (p. 199) (Freud, 1940/1964). The secondary process allows for a delayed and controlled discharge of energy, as for example realized by making plans and anticipating consequences (Schimek and Goldberger, 1995). The directing, binding, or investing of primary process energy is referred to as *cathexis*^2^ (Ornston, 2002; Holder, 2014). An object or event that is cathected by the ego –be it physical, social, or intrapsychic– becomes invested with an *affective charge* and mentally represented as exhibiting a libidinal, motivational, emotional, and/or bodily relatedness to the own person (McGlashan, 2009; Northoff, 2011). The primary process is psychometrically assessable and has been studied in altered states of consciousness like dreams, psychosis, anxiety, and the psychedelic experience (Auld et al., 1968; Brakel, 2004; Bazan et al., 2013; Kraehenmann et al., 2017).

### 2.2. Ego psychology

*Ego psychology* has its roots in Freud’s structural model. Unlike Freud, however, who saw the development of the ego as conflict-derived and governed by drives, ego psychology stresses the ego-inherent adaptive capacities (Schamess and Shilkret, 2008; Auchincloss and Samberg, 2012). Ego psychology represents one of the major theorems of psychoanalysis (Pine, 1990) with various branches (Marcus, 1999) and modern developments (Busch, 1995; Richards and Lynch, 1999; Eagle, 2021). Important contributions include the systematization of ego defenses (Freud, 1942), the concept of ego strength (Fenichel, 1938/1954; Nunberg, 1942), Erikson’s (1998) model of lifelong ego development, the operationalization of major ego functions (see Sections 2.2.1–2.2.3), as well as the elaboration of ego-centered forms of therapy (Blanck and Blanck, 1974; Busch, 1995). The ideas of Heinz Hartmann, in particular, have primed a modern understanding of the ego with emphasis on its capacity for synthesis and mastery, rather than subjugation to id and the outer world (e.g., Palombo et al., 2009a).

Hartmann’s (1958) ego psychology suggests that motility, perception, memory, and intelligence are apparatuses of an inborn ego constitution that provide an individual with a basic equipment for adaptation to an *average expectable environment.* The ego-inherent apparatuses (also referred to as *primary autonomy*) are part of the human heritage, “cannot be traced […] to the influence of the instincts and of reality” (p. 167) (Hartmann, 1964), and at least to begin with exist in a *conflict-free sphere* uncorrupted by id frictions (Hartmann, 1958). As “precursors of what will later be specific ego functions” (p. 236), they develop into to what Hartmann (1964) calls the “powerful triad of [ego] functions: adaptation, control, and integration (synthetic function)” (p. 290). The latter is sometimes referred to as the core function of the ego and will further be discussed in the next section. Although Hartmann’s (1953) theory assumes that the inborn ego apparatuses have their own pool of primary energy, the maintenance of the ego functions still requires energy derived from neutralization of (aggressive and libidinal) drives (Hartmann, 1964). Upon failure of neutralization, the ego is deprived of primary process energy, which according to Hartmann (1953) gives rise to the functional *ego disturbance* characteristic of acute states of psychosis (Bellak et al., 1973). Symptoms of psychedelic drug action have been interpreted along similar considerations (see Section 4.1.1).

#### 2.2.1. Ego functions – synthesis and the sense of self

As one of the most systematic approaches up to date, Bellak et al. (1973) have operationalized a taxonomy of 12 ego functions (Table 1), which they tested for reliable discrimination of healthy, neurotic, and schizophrenic subjects –and later also of borderline characters (Bellak and Goldsmith, 1984). Conceptually, Bellak’s ego functions are largely rooted in the tradition of drive and ego psychology. *Autonomous functioning,* for instance, accommodates Harmann’s *primary autonomy* concept. Different functions of the ego may evolve at different stages of a person’s life cycle, and in friction with life phase-dependent developmental tasks (Loevinger, 1976b; Erikson, 1980, 1987; Tyson and Tyson, 1990). Various ego psychologists emphasize the importance of the ego’s synthetic function (Nunberg, 1931; Hartmann, 1958; Blanck and Blanck, 1979), referring to its capacity to bring together divergent processes of the psyche and form a coherent organization. Along these lines, for instance, a coherent *sense of self* can be considered as a downstream product of the synthetic function of the ego. The self, as defined by Hartmann (1950), refers to the own person in distinction to the environment. The ego can generate a mental representation of the outer world, as well as of the own person (object- vs. self-representation). A coherent sense of self arises as the ego gradually incorporates (or synthesizes) various of such self-representations into a supraordinate sensory-mnestic structure of who the own person is (Kernberg, 1982). Loevinger (1976a) understands synthesis as the *essence of the ego*. It “is not just another thing the ego does, it is what the ego is” (p. 5). Thinking of the ego as a joint between functions, rather than the functions themselves is an important conceptual nuance, which will be readdressed when considering the neurobiological correlates of psychedelic-ego interaction (Section 3.2).

**Table 1:** Ego functions and their components, as defined by Bellak and Sheehy (1976).

Ego functions	Components
Reality testing	Distinction between inner and outer stimuli
	Accuracy of perception of external events (incl. orientation to time and place)
	Accuracy of perception of internal events
Judgment	Anticipation of consequences of intended behavior
	Manifestation of this anticipation in behavior
	Appropriateness of behavior to external events
Sense of reality	Extent of derealization
	Extent of depersonalization
	Self-identity and self-esteem
	Clarity of boundaries between self and world
Regulation and control of drives, affects, and impulses	Directness of impulse expression
	Effectiveness of delay mechanisms
Object relations	Degree and kind of relatedness (narcissistic attachment or symbiotic object choices)
	Primitivity versus maturity
	Degree to which others are perceived independently of self
	Object constancy
Thought processes	Memory, conception, and attention
	Ability to conceptualize
	Primary versus secondary process as reflected in communicative language
Adaptive regression in the service of the ego (ARISE)	Ability to regressively relax cognitive acuity
	Ability to allow new configurations to emerge in thinking
Defensive functioning	Weakness or obtrusiveness of defenses
	Success or failure of defenses
Stimulus barrier	Threshold for stimuli
	Effectiveness of management of excessive stimulus input
Autonomous Functioning	Degree of freedom from impairment of primary autonomy apparatuses
	Degree of freedom from impairment of secondary autonomy apparatuses
Synthetic-Integrative Functioning	Degree of reconciliation of incongruities
	Degree of active relating together of events
Mastery-Competence	Competence (how well a person performs in relation to his or her capacity to actively master and affect his or her environment)
	Feeling of competence as measured by person’s expectations of success on actual performance
	Discrepancy between actual competence and feeling of competence

#### 2.2.2. Ego functions – ego boundary

The *ego boundary* is one of the ego functions most often discussed in contemporary psychedelic research (Roberts and Winkelman, 2013; Preller and Vollenweider, 2016; Smigielski et al., 2020; Scheidegger, 2021; Fischman, 2022); conceptually similar terms include *psychic membrane*, *psychic envelope,* or *contact barrier* (Rabeyron, 2021). *Ego boundary* was first introduced by the Freud disciple Tausk (1919, 1933), and later adopted by Federn (1952) to explain certain symptoms of schizophrenia. The internal ego boundary separates the ego from unconscious material, hence, overlapping with the concept of *defense mechanisms* (see next Section). The external ego boundary separates a person from his or her environment but does not necessarily coincide with physical body boundaries (Landis, 1970). Examples like the rubber hand illusion, body swap phenomena (Solms and Panksepp, 2012), or psychedelic disembodiment (Silverstein and Klee, 1958) demonstrate how external boundaries can expand or weaken, leading to overlapping representations of self and objects. Intact ego boundaries are essential for *reality testing* and a sustained *sense of reality* (Federn, 1952) and have been conceptualized in the context of *ego cathexis* (Jacobson, 1954). Reality testing (see Table 1) requires the ego to match ongoing perceptual input across different senses and compare the resulting multisensory integral with mental representations of past percepts (Bellak and Goldsmith, 1984). In such a matching process, there only seems to be a thin line for the ego to cathect a perceived object as being internal or external to the own body. Conflicting multisensory input, for example, and/or expectancy effects enforced top-down by mental representations of the own body may be sufficient to have the ego miscathect a rubber hand as being part of the bodily self (e.g., Mandrigin and Thompson, 2015). Difficulties in cathecting the physical enviroment (including the own body and own thoughts) as externally or internally related (or, on the opposite, as unrelated) to the self, are likely to underlie some of the symtpoms of the psychedelic experience and will be therefore readdressed in Section 4.1.1.

#### 2.2.3. Ego functions – ego defenses and character

Defense mechanisms are vital ego functions that operate mostly unconsciously to protect against experiencing psychological discomfort, for instance caused by conflict. They defend against internal sources of excitement –such as drives or unpleasurable affects– and representations associated with them (e.g., wishes, fantasies, or memories), aiming to maintain the equilibrium and psychological integrity of a person (Laplanche and Pontalis, 1988). Defenses differ from coping strategies, which are consciously employed. Psychometric instruments have been developed to assess defenses and validate them across disciplines (Cramer, 2006, 2015). Vaillant (1977, 1995) proposed a hierarchy of defenses based on a long-term study, categorizing them by their level of maturity (see Table 2), which now has been widely adopted (rev. Safyer and Hauser, 1995; Cramer, 2006).

**Table 2:** Hierarchy of ego defenses as ordered by their level of maturity (non-exhaustive list).

Level of maturity	Mechanisms	Definition
Psychotic	Delusional projection	Frank delusions about external reality, usually of a persecutory type, with abandonment of reality testing
		Perception of one’s own feelings in another person and/or perception of other people or their feelings inside oneself
	Distortion	Grossly reshaping external reality to suit inner needs (incl. unrealistic megalomaniacal beliefs, hallucinations, wish-fulfilling delusions); usually, unpleasant feelings are replaced with their opposites
Immature	Passive-aggressive behavior	Aggression toward others expressed indirectly and ineffectively through passivity or directed against the self (e.g., via procrastination)
	Acting out	Direct expression of an unconscious wish or impulse (e.g., via chronic drug abuse or self-inflicted injury) to avoid (a) being conscious of the affect that accompanies it, and (b) the tension that would result from postponement of instinctual expression
Neurotic	Intellectualization	Thinking about instinctual wishes in formal, affectively bland terms, and not acting on them; the idea is conscious, but the feeling dismissed
		Includes paying attention (1) to the inanimate in order to avoid intimacy with people; (2) to external reality to avoid expression of inner feelings; (3) to irrelevant detail to avoid perceiving the whole
	Reaction formation	Behavior diametrically opposed to an unacceptable instinctual impulse (e.g., overtly caring for someone else when one wishes to be cared for)
Mature	Suppression	(Semi-)Conscious decision to postpone (but not avoid) paying attention to a conscious impulse or conflict
		Includes looking for silver linings or minimizing acknowledged discomfort
	Sublimation	Indirect or attenuated expression of instincts without either adverse consequences or marked loss of pleasure (e.g., expressing aggression through pleasurable games, sports, and hobbies)
		Unlike with neurotic defenses, instincts are channeled rather than dammed or diverted

Adapted from Vaillant (1977). Less mature defenses distort reality more severely and are therefore more costly to the adaptability of the ego. A mature character allows the ego to flexibly recruit defenses from all levels; a less mature character confines the ego to low-level defenses.

An individual usually has a preferred set of defenses, which he or she habitually employs as a part of their *character*. The discourse on *character* as a psychological construct has a long history (e.g., Roback, 1931). In psychodynamics theories, character either refers to the manifest or the latent level of a person’s habitual functioning –the behavioral and attitudinal patterns visibly displayed to the outside world and the psychological processes inferred to underlie the given patterns, respectively (Baudry, 1995). Ego-psychologically, the defensive component of character has been likened to a protective *hardening* or an *armor* of the ego (Reich, 1972). In a broader sense, however, character comprises not only the ego’s habits of defense but also its overall “habitual mode of [synthetically] bringing into harmony the tasks presented by internal demands and by the external world” (p. 427) (Fenichel, 1945). Character, hence, represents the style, preference, or consistency of how the ego executes its functions over time (Prelinger and Zimet, 1964) –this includes intrapsychic conflict management, self-regulation, environmental adaptation, and relating to others (Auchincloss and Samberg, 2012). The close relationship between the ego and the character is crucial, as the ego operates within the constraints set by the character. Limited, rigid, or immature use of defenses, for example, can be indicative of character pathology and compromise the ego’s adaptability. *The Psychodynamic Diagnostic Manual* (PDM2) (Lingiardi and McWilliams, 2017) identifies 12 character types or styles,^3^ which for the most part are listed as disorders by DSM5 and ICD10 (Widiger, 2012) but may also occur among healthy individuals (Oldham and Morris, 2012; Sachse, 2019). Examples include the obsessive, the histrionic, the narcissistic, and the schizoid type. A person’s character influences how he or she reacts to psychedelic drugs (Barr et al., 1972; Grof, 1980a) and might give important diagnostic clues about central sites of *ego hardening* and potential targets for psycholytic and other forms of psychedelic-assisted therapy (see Section 5.2).

### 2.3. Alternate ego states

#### 2.3.1. Ego regression

*Regression* was introduced to psychodynamic parlance by Freud (1897/1966, 1900/1953) and received one of its most important connotations along Freud (1913/1958) and Abraham’s (1924/1988) theory of child development –the stages of which they associated with certain intra- and interpersonal conflicts. If unresolved, so their theory suggests, a developing ego may stay fixated and in later life tend to gravitate (thus show an inclination for regression) toward interpersonal themes and behavioral tendencies of the unresolved stage (Palombo et al., 2009b). Regression can be defined as “a re-emergence of modes of mental functioning characteristic of earlier phases of psychic development” (p. 82) and assume various degrees with primitive modes often co-existing side by side with more mature forms (Arlow and Brenner, 1964): Regression can be adaptive and act in the service of the ego. It can be recruited as a defense mechanism or for diagnostic and therapeutic purposes, but also represent a concomitant of psychopathology (Leiper and Maltby, 2004; Auchincloss and Samberg, 2012; Maroda, 2012). Regression in service of the ego, for instance, contributes to religious experiences (Fauteux, 1994) and allows the symbolism of the primary process to be expressed in the form of art and poetry (Kris, 1952). Psychodynamically oriented long-term therapies usually aspire elements of what Balint (1968) called *benign regression* or *regression in the service of progression*. “Going back to something ‘primitive’, to a point before the faulty development started […], and […] at the same time, discovering a new, better-suited, way which amounts to a progression” (p. 132), Balint argues, paves the way for therapeutic changes in the ego in consequence of a “shed[ing] [of] all sorts of character and defensive armours” (p. 135) (Balint, 1968). Regressive elements of such benign or progress-oriented character are likely to constitute key components to psychedelic healing and will be discussed in more detail below (see Sections 3.1.2 and 5.2).

#### 2.3.2. Ego disturbance

*Ego disturbance* is another descriptor of altered ego functioning. In contrast to ego regression, however, it is a nosological term generally implying mental pathology. The term *ego disturbance* (German, *Ich-Störung*) originated outside of psychodynamic theories in general psychiatric parlance describing schizophrenic symptoms (Gruhle, 1915; Schneider, 1950/1976; Buergy, 2011). Ego psychologists initially associated ego disturbance with character pathology and maladaptive use of defenses (e.g., Fenichel, 1938) and nowadays intertwine character pathology with concepts of psychosis (e.g., Marcus, 2017; Kernberg, 2019). In contemporary German-tradition psychiatry, ego disturbance is understood as a syndrome encompassing various symptoms related to psychosis (see Table 3). For example, depersonalization involves perceiving oneself as alien or estranged (Broome et al., 2017) and entails an ego split “into a part which feels estranged and one which carries on the observer’s role” (p. 762) (Stamm, 1962). Psychosis, particularly acute forms of schizophrenia (rev. Munich, 1995; De Masi, 2018), is often considered as a prototype of an ego disturbance (Weiner, 1966) with broad-spectral impairments in ego functioning (Bellak et al., 1973), immature defenses (Cramer, 2006), and primary process thinking (Bazan et al., 2013). Impairments in *reality testing* during psychosis, for instance, with an anxiously experienced self-environment indistinction (Benedetti, 1983; Eigen, 2004) is often interpreted as a state of ego regression to a primordial phase in development where the symbiosis with the mother has been unresolved yet (Mahler, 1952).

**Table 3:** Symptoms of ego disturbance as defined by the *manual for assessment and documentation of psychopathology in psychiatry* [adapted from Broome et al. (2017)].

Symptom	Definition
Boundary disturbance	Disturbance in the perception of the self as distinct from the environment, or of the integrity of the self
Derealization	Surrounding or time are experienced as if they were changed; feelings of familiarity with and trust in the environment are lost
Depersonalization	Perception of oneself as alien, unreal, changed, or as a stranger
Thought broadcasting	Personal thoughts are experienced as no longer belonging to oneself alone but accessible to others
	May be experienced as passive, that is others are not perceived as actively attempting to read one’s mind
Thought withdrawal	Personal thoughts are experienced as being removed or stripped from oneself
Thought insertion	Personal thoughts and ideas are experienced as externally influenced, made, controlled, directed, entered, or imposed
Passivity	Feelings, intentions, behavior, and/or bodily functions are experienced as externally controlled or made by others

The psychedelic state and acute forms of psychosis share several symptoms of ego disturbance (Hermle and Kraehenmann, 2018; Friesen, 2022). Not only does this make psychedelic drug action one of the most often referred to pharmacodynamic models of endogenous psychosis (Aghajanian and Marek, 2000; Vollenweider and Geyer, 2001; De Gregorio et al., 2016), but it also allows us to put some of the ego-theoretical concepts on psychosis into the service of understanding psychedelia (see Section 4.1.1.).

## 3. The ego in psychedelic drug action

*Ego disturbance* was early on suggested as “the general effect of mescaline intoxication” (p. 83) (Buchanan, 1929) and the defining characteristic of the LSD experience (Weyl, 1951). Klee (1963) noted that it was in fact quite common for the literature of his time to state that the psychedelic state involves functional ego impairments. “Although such statements are commonly encountered,” he stated, “in very few instances are they documented in anything but an anecdotal manner” (p. 462). Trying to bridge the empirical gap, Klee (1963) analyzed data on the mental effects of LSD from hundreds of healthy volunteers, and observed a variety of symptoms that speak in favor of psychedelic ego alterations. These include depersonalization and derealization, leakiness of the stimulus barrier and the ego boundary, as well as regressive tendencies toward primary process thinking and less mature defenses. Klee related his observations to the conceptual framework of ego psychology and emphasized the “usefulness of this theoretical model in understanding the effects of LSD” (p. 69) (Klee, 1963). Hermle et al. (1988), who analyzed own clinical material and experimental protocols of Beringer (1927) likewise provided evidence for psychedelic-induced ego alterations. Based on these observations, the following paragraphs review scientific evidence supporting the idea that psychedelics affect key aspects of the psychological ego process. Additionally, preliminary considerations on the underlying neurobiological mechanisms are presented. Aim is to discuss the ego-construct as it is used in contemporary psychedelic research and build a bridge to the subsequent understanding of psychodynamic models of the psychedelic experience and therapeutic implications thereof from an ego-theoretical perspective.

### 3.1. Psychedelic depersonalization, derealization, and ego dissolution

In psychedelic research, depersonalization, derealization, and ego dissolution are commonly mentioned symptoms of ego disturbance, to be found in the earliest publications already (Bromberg and Tranter, 1943; Savage, 1952; Szara, 1957; Delay et al., 1958). Depersonalization and derealization are concomitants of various psychopathologies, where they occur to varying degrees and with varying emotional discomfort (Coons, 1996; Simeon and Abugel, 2023). In line with modern terminology (see Table 3), mescaline-induced depersonalization for example is described as a feeling of alienation from the own person that may progress toward a self-loss and cosmic unity (Guttmann and Maclay, 1936). Derealization is characterized as the experience of familiar surroundings appearing strange and devoid of their usual emotional relatedness to the own person (Guttmann, 1936). Similarly, LSD-induced depersonalization and derealization come with a loss of emotional connection to the own body and the body-external environment, respectively (Becker, 1949). Depersonalization and derealization undermine the ego’s ability to *self-relate* objects, that is to make the objects carry an affective charge that signifies their relation to the own person (compare Section 2.1.1) (e.g., Sarlin, 1962). Ego-psychologically, therefore, the psychedelic experience can be conceptualized as reflecting a process of *decathexis*– an idea further discussed in Section 4.1.1.

In concurrent research (e.g., Gouzoulis-Mayfrank et al., 2005; Studerus et al., 2011; Schmid et al., 2015), depersonalization, derealization, and ego dissolution are often uniformly quantified by means of the *5-Dimensional Altered States of Consciousness* (5D-ASC) questionnaire. If emotionally positively experienced, they are covered by the *oceanic boundlessness* scale; if negatively experienced, by the *dread of ego dissolution* scale (Dittrich et al., 2010). Depersonalization and derealization may be precursors to more comprehensive processes of disintegration, dissolution, loss, or even death of the ego. Terminologically, there does not seem to be a general consensus, though, and *ego* and *self* are often interchangeably used in the field –as exemplified by the *Ego-Dissolution Inventory* item “I experienced a disintegration of my ‘self’ or ego” (Nour et al., 2016). One might suggest that ego disintegration refers to a breakdown of the integrative core function of the ego so that various of its downstream functions can no longer be synthesized into an overall organization. Central to ego dissolution, as opposed, seems to be the disintegration of its boundary function and the associated blurring of self-object distinction (Nour et al., 2016; Fink, 2020; Martial et al., 2021). Figuratively speaking, ego dissolution might be imagined as a centrifugal dispersal from a concentrate of self-representations that gradually radiates until in ego death no centripetal core, no concept or frame of reference is left for the ego to invest in or orbit around. Aptly, Grof (2000) defines ego death as “death of our old concepts of who we are and what the world is like” (p. 52). As such, ego dissolution is conceptually in line with a regression to an infantile ego organization, that is marked by representational indistinction and freedom from the usual ego patterns as determined by a person’s character.

#### 3.1.1. Psychedelics, ego remnant, and contact to reality

Depersonalization, derealization, and a blurring of ego boundaries: Psychedelics undoubtedly interfere with those ego functions that primarily anchor a person to reality (see Table 1). Surprisingly, true hallucinations and complex delusions are not typically associated with classic serotonergic psychedelics at common doses (e.g., Preller and Vollenweider, 2016). Insight into the situational context, with sustained attribution of one’s state to drug intake, indeed differentiates serotonergic psychedelics from anticholinergic delirants (e.g., Hollister et al., 1960; Fuxe et al., 1976) and only gradually drops as a function of escalating doses (Isbell et al., 1956; Klee et al., 1961; Leuner, 1962a).

Early on, Becker (1949) noted that the ego under the influence of LSD withdraws from experiencing, enacting, or embodying the own person while continuing to observe and compare its representation to the pre-drug state. This dissociation between experience and observation was also noted by other researchers (e.g., “the observing ego faces the observed self in a […] detached way” [p. 211]) (Guttmann, 1936; Linton and Langs, 1964). Leuner (1962a) refers to the remaining observer as a *reflecting ego remnant* and suggests that its failure may explain why the artificiality of the psychedelic experience becomes less appreciable in psychotic courses of high-dose experiences. The inability to relate one’s current state to a time-enduring self-representation is thought to distinguish psychotic and borderline individuals from those with more mature, reality-oriented characters (Caligor et al., 2018). Hence, a psychedelically weakened ego with impaired self-object distinction may still maintain contact to reality as long as it can generate a self through observation, compare it to past self-representations, and attribute any differences to the drug effect. The prevalence of an observing ego faculty is a prerequisite for any psychotherapeutic intervention (Busch, 1995) and therefore an important clinical feature of the psychedelic experience.

#### 3.1.2. Psychedelics, regression, and ego defenses

Most therapists of the first-wave psychedelic research agree that the psychedelic experience is psychodynamically conceivable as a regression to a less integrated ego organization (Sandison, 1954; Savage, 1955; Arendsen-Hein, 1963; Ward, 1967; Grof, 1968; Berendes, 1980). Cutner (1959), for instance, stated that LSD is “instrumental in producing a state of regression to a phase of development before the ego was strong enough to cope with the id-forces.” “Under LSD,” she continued, “the ego-threshold is […] lowered and the patient’s defenses against the impact of emotional and instinctual […] contents are […] weakened” (p. 722). Functional regression with recapitulation of different developmental stages of a person’s life may occur (Fernandez-Cerdeno and Leuner, 1965; Adler, 1981). Although such processes are difficult to objectify, verbal reports, formal analysis of vocabulary used in written reports (Martindale and Fischer, 1977), paintings (Leuner, 1963), frank infant-like behavior (Cattell, 1957; Grof, 1975), as well as immature performance in perceptual and intellectual capacity tests (Wapner and Krus, 1960; Lienert, 1966) during or in retrospection of the psychedelic experience speak in favor of regressive elements.

Different ego defenses seem to show different sensitivity toward LSD, making people of different character types differentially susceptible to psychedelic regression (Barr et al., 1972). The obsessive type, for example, has particularly rigid ego defenses with a character-inherent tendency for general affect suppression, and appears to require high doses to overcome his or her characterological and/or constitutional resistance to psychedelics (Leuner, 1962a; Grof, 1975). The relationship between ego defenses, regression, and character is central to the psycholytic approach of psychedelic healing and will be readdressed in Section 5.2.

### 3.2. Neurobiology of ego alterations – a matter of network (de-)synchronization?

The functions attributed to the ego are psychologically widespread (see Table 1). Rather than understanding the ego as represented by those brain regions that mediate individual of these functions, however, it appears more plausible to think of the ego as the process that orchestrates the engagement or disengagement of the brain regions involved (compare Section 2.2.1). The frontal lobe, for instance, is a major association hub that is often discussed regarding (ego-like) executive functions (Reitan and Wolfson, 1994). Indeed, a major line of research speaks in favor of a mechanistic role of the (pre-)frontal cortex in the neurophysiology of psychedelic drug action (Hermle et al., 1992; Vollenweider et al., 1997; Riba et al., 2006; Buchborn et al., 2015; Marek, 2017; Mason et al., 2020).

Not all ego functions are directly executive, though. The ego’s defensive apparatus and the stimulus barrier, for instance, work out of a person’s awareness and rather build a foundation for the executive functions of the ego to work frictionlessly. Psychedelics interfere with preattentive sensorimotor gating (Halberstadt and Geyer, 2018), a filter process conceptually reminiscent of the ego’s stimulus barrier function (Bellak et al., 1973). The psychedelic-induced filter deficit appears to have roots in the locus coeruleus (Rasmussen and Aghajanian, 1986), the pallidum (Sipes and Geyer, 1997; Ou et al., 2023), and/or in a thalamic filter deficit (Geyer and Vollenweider, 2008; Preller et al., 2019). If the brain areas underlying the psychedelic stimulus barrier disruption are so widespread already, a more globally induced ego disintegration is not likely to emerge from interference with a singular brain hub either but rather from network activity or the underlying (de-)synchronization processes. Hence, the next paragraphs review first neurobiological research on psychedelic network activity and its relation to the psychodynamic ego and the ego-id duality, respectively.

#### 3.2.1. Psychedelics, ego-id duality, and the anarchic brain hypothesis

Up to date, only Carhart-Harris and Friston (2010, 2019) have pioneered into the neurobiological correlates of psychedelic drug action accounting for the psychodynamic ego. Conceptualizing the ego in light of synchronization processes, they suggest that the ego is neurobiologically represented by the *default mode network* (DMN) –a high-level brain organization consisting of the medial part of the frontal cortex, the medial temporal lobe (MTL) and others (Whitfield-Gabrieli and Ford, 2012). Functional integrity of the ego, according to their model, is represented by synchronous oscillatory activity within the DMN, including the alpha-frequency band (Carhart-Harris et al., 2014).

In line with Freud’s ego-id duality, various early authors suggest that the ego disintegration characteristic of the psychedelic state is complemented by one major additional factor, namely arousal or labialization of affects (Becker, 1949; Leuner, 1962a; Klee, 1963). A two-factor structure of the psychedelic state is also reinforced by a more recent psychometrical approach (Lebedev et al., 2015). Psilocybin and LSD reduce the amplitude of alpha oscillations in the cortex (Muthukumaraswamy et al., 2013; Carhart-Harris et al., 2016), which is thought to favor emotion- over knowledge-based modes of thinking (Klimesch, 2012). Additionally, psilocybin decouples the MTL (which includes [para-]hippocampal limbic structures) from the rest of the DMN. This leads to a disinhibition of MTL activity (Carhart-Harris et al., 2014; Tagliazucchi et al., 2014), possibly affecting other components of the limbic system as well (Monroe et al., 1957). Along the *anarchic brain hypothesis* (Carhart-Harris and Friston, 2019), this is interpreted as the ego-governed secondary process losing control over the affect-dominated primary process of the id (Kraehenmann et al., 2017).

#### 3.2.2. Psychedelics, hallucinatory discharge, and the REBUS model

As the ego withdraws, more archaic content can enter the field of experience. The REBUS (relaxed beliefs under psychedelics) model suggests that experience-based expectations about the sensory organization of the world (*priors*) are represented by the DMN and largely structure in which way a given input is allowed access to consciousness (Carhart-Harris and Friston, 2019). Cortical top-down structuring of sensory input by the DMN works along principles of *hierarchical predictive coding* and is suggested to be a major correlate of ego related secondary processing. Its task is to minimize free energy related to sensory ambiguity by making inferences about possible causes of a given input based on expectation and belief. If cortical top-down structuring fails, so the REBUS model, sensory input may come in as ambiguous and leave its processing up to mere bottom-up appraisal by the limbic system (Carhart-Harris and Friston, 2010, 2019). Here, sensory impressions and emotional states are not bound by the ego via primary-to-secondary process translation. They remain unthinkable, uncontained, and are allowed premature entrance to consciousness for the sake of immediate discharge (e.g., via [pseudo-]hallucinatory visuals). Such discharge has important therapeutic implications and will further be discussed in Sections 4 and 5.

#### 3.2.3. Psychedelics, ego boundaries, and cross-network synchronization

Psychedelic-induced disembodiment phenomena (Savage, 1955; Silverstein and Klee, 1958; Ho et al., 2020) can be seen as indicative of an increased permeability of the external ego boundary. The DMN engages in internally oriented mental processes like introspection and daydreaming and disengages when a so-called task-positive network of the brain reorients the attention from within to stimuli of the outside world. Psychedelics reduce the orthogonality between both systems allowing them to functionally couple with one another. Since a temporal overlap between internal and external representations is reminiscent of a melting of the external ego boundary, the given cross-network coupling has been discussed as a possible neurobiological correlate (Carhart-Harris et al., 2013; Stoliker et al., 2022).

## 4. Psychedelic psychodynamics

During the first wave of research on the psychotherapeutic potential of psychedelic drugs, from the 1960ies to the 1970ies, two approaches dominated the field –the psychedelic therapy, mostly performed in the USA, and the psycholytic therapy with predominate seat in Europe. In the psychedelic approach, over one to three sessions, higher doses of psychedelics are applied to patients with the aim to induce overwhelming “conversion-like existential experiences” (p. 13) that shake the patient from the core and thereby invite changes in character, motivation, and/or belief system. The psycholytic approach, on the other hand, applies lower doses of psychedelics over 10 to 50 sessions and facilitates psychodynamically oriented forms of therapy. Primary aim is character maturation, which among others involves (re-)integration of self-object dynamics, for instance via “loosening of infantile parental bonds” (p. 13) (Passie, 1997b). Major representatives of the psycholytic paradigm include Ronald Sandison in the UK, Hanscarl Leuner in Germany, and more recently the *Swiss Physicians Society for Psycholytic Therapy* (SÄPT). A combination of both approaches, with implementation of transpersonal elements, has been advocated for by Stanislav Grof (rev. Passie, 2021).

### 4.1. Psychodynamically oriented models of the psychedelic experience

Before now turning to the therapeutic implications of psychedelic drug action, specifically accounting for the psychodynamic framework of psycholytic therapy, it is important to integrate some of the above reviewed ego related concepts and understand how they may help and inform on the dynamics of the psychedelic experience.

#### 4.1.1. Psychedelia as a state of progressive decathexis

Savage (1955), an early psychiatrist at the *US National Institute of Mental Health* in Maryland, conducted 300 clinical observations on 38 subjects to develop his understanding of the psychodynamics of LSD. He applied the concepts of *ego cathexis* and *ego regression* to his analysis. According to Savage, LSD enhances affect arousal and initially the ego’s cathexis thereof. With an increase in cathexis –that is the process by which the ego affectively interrelates and/or embodies a physical object or event of its environment– one’s sensitivity to bodily sensations, one’s alertness to environmental stimuli, one’s thought production, and one’s richness of ideas become amplified. Over time, cathexis is gradually withdrawn, leading to an estrangement from one’s body and one’s body-external surrounding (i.e., depersonalization and derealization); likewise, identification with one’s thoughts and feelings becomes impaired. Positive thoughts may feel revelatory, while negative thoughts may seem alien or accessible by others. Based on ego-theoretical concepts of psychosis, Savage (1955) suggests that LSD primarily disrupts the availability of mental energy to the ego, undermining its ability to align perceptions with memorized representations (compare Sections 2.2, 2.2.2, and 2.3.2). The ego’s failure to adequately invest in representations, including those of the own person, may result in a fragmented sense of self –a conceptualization well in line with disruption of top-down priors as posited by the REBUS model (Carhart-Harris and Friston, 2019). Here, with an unsteadiness of the ego’s cathectic relation to the self, the ego enters a “condition in which various ego states succeed one another without a common reference point” (p. 78) (Fischman, 1983) and eventually ends up in regressive early-life state where self and object were marked by symbiotic indistinction.

Whether such a state has the potential for therapeutic effects or not is dependent on set and setting, including the autobiographic and/or symbolic content that is released by the regression, and the sustainability of the client-therapist alliance –which we address in the following sections.

#### 4.1.2. Psychedelia as a state of inner stimulus overdrive

Leuner (1962a,b), a pioneer of psycholysis in Germany (Passie, 1997a), based his model of psychedelia on over 1,000 clinical observations involving psilocybin, LSD, and anticholinergics. Congruent with the ego-id duality outlined in Section 3.2.1, Leuner (1962a, 1968) identified two structural changes of the psyche as hallmarks of the psychedelic state: A regression of ego functions to an ontogenetically earlier stage marked by passivity, concretistic thinking, and an inability to keep the stream of consciousness organized and unfragmented; and an excitation of inner stimulus production, affect overdrive, and a tendency to discharge the accumulating excitation via motor stereotypes, (pseudo-)hallucinations, vivid early-life reminiscences, and/or fantasies: “The psyche is no longer able to dam up the activated id structures via the [internal] ego [boundary]” (translated from German, p. 45) (Leuner, 1962a). The content of the resulting experience is determined by emotionally themed memory constellations, so called *transphenomenal dynamic governing* (tdyst) systems. Therapeutically relevant tdyst systems often evolve around repressed negative life events, whose affective dynamics have remained undischarged. Being tense and affectively loaded, they seek for expenditure, and therefore utilize the drug induced weakening of ego defenses to get expended along the internal stimulus overdrive. In this model, psychedelic (pseudo-)hallucinations are symbolic of an underlying tdyst system (Leuner, 1962a; Passie, 2005). They can be understood as a direct expression of instinctual demands, as a projective externalization of punitive (parental) objects, or in service of the ego as restitutionally invoked defenses or first efforts to integrate the underlying life events (Pilowsky, 1986).

#### 4.1.3. Psychedelia and the psychodynamic realm

Grof (1968, 1975), who used clinical material from more than 1,100 LSD and psilocybin sessions, integrates psychodynamic and transpersonal concepts and within his *cartography of the human mind* distinguishes four realms of psychedelic experience: (1) The abstract and esthetic realm characterized by drastic perceptual changes, most strikingly “orgies of vision” (p. 40); (2) the psychodynamic realm derived from ego regression and re-enactments of emotionally charged life events; (3) the birth-and-death centered perinatal realm with intrauterine-like feelings of cosmic unity, ontological crises, and ego death; as well as the (4) transpersonal realm in which the ego is thought to transcend body, time, and space (Grof, 1975; Grof and Halifax, 1977). The psychodynamic realm is determined by so-called *condensed experience* (COEX) systems (Grof’s analog of Leuner’s tdyst systems), which contain repressed memories of various life events and/or fantasies interlaced by a common emotional quality or theme. The confrontation with such a memory constellation bears significant therapeutic potential: The affective load bound to it can be abreacted (or discharged), the underlying life events relived, and the theme behind it (often a conflict) understood (Grof, 1968).

## 5. Discussion

### 5.1. Summary and synthesis

In this paper’s theoretical framework, we understand the ego as a synthetic process that organizes and executes a set of different functions in service of the best possible adaptation of a person to their changing environments. The ego’s time-sustained or habitual modes of organization and execution are referred to as character. As to the ego’s relation to the id, we simplistically think of a duality between higher cognitive and structuring functions as opposed to the rather unstructured dynamics of affectivity (including instincts, emotions, and motivation). Affectivity can energetically drive the functions of the ego, but also overwhelm its resources. Central to the ego is its capacity to synthesize divergent or conflicting aspects within and across the psyche. This includes its capacity to generate an internal representation of the own person (i.e., a sensory-mnestic self-representation) that is continuous across varying situations and that is distinct from the representation of the environment. We assume that psychedelics interfere with the functional integrity of the ego, which shifts the ego-id equilibrium toward the id and toward unbound affectivity. Due to partial ego failure, the continuity of self-representations is no longer reinforced by adequate sensory updates, or the updates are no longer brought in congruence with the memorized representations. The ego thereby loses its major frame of reference: It becomes more fluent in identifying with alternate self-representations, or even includes the environment –hence, dissolving ego boundaries. The ego may now be regressively pulled to developmental points of fixation, to earlier life events of strong affective charge and unresolved conflict. Here, the ego may find a more primordial frame of reference and adopt a level of ego functioning characteristic for this early-life period; this includes less mature ego defenses like hallucinatory projection. In this paper’s theoretical framework, it is this very scenario of a regressed ego state, wherein the seed for psycholytic healing is sowed.

### 5.2. Therapeutic implications – principles of psycholytic healing

In line with Grof’s model of the *psychodynamic realm* (see Section 4.1.3), Leuner (1963) stresses four major principles of psychedelic-assisted, psycholytic psychotherapy: (1) The regressive recapitulation of traumatic or frustrating biographic events along loosened ego defenses with release of unconscious material and discharge (or *cathartic abreaction*) of event-related affective load; (2) insight and understanding of how the original traumatic events, and associated conflicts, have unconsciously lingered on into adulthood and created maladaptive, rigidified ego defenses and an impaired capacity to emotionally relate to others; (3) a facilitation of the so-called *transference* process, through which the client perceives or emotionally relates to the therapist as if he or she was the person originally involved in the traumatic event (e.g., the abusive father); and (4) a meaningful *synthesis* that integrates the conflicts of earlier life events and assimilates their now *disarmed* affective dynamics into a more flexible character structure. If spontaneously occurring during the psychedelic experience itself, *synthesis* is experienced as sudden rise in strength potentially culminating in a mystic-type experience – the *unio mystica* or oneness (Leuner, 1962a, 1963). Unlike psychedelic therapy, however, psycholytic therapy does not commonly aspire a (full) disintegration of the ego. The defenses of the ego as well as the ego’s (cathectic) relation to the self are weakened but maintained (Passie, 2015; Passie et al., 2022).

#### 5.2.1. Character – a compass to psycholytic regression?

If we assume that the ego is the primary target of every psychological intervention (Busch, 1995) and that all changes in perspective and behavior of a person can only be adopted and put in action through the functions of the ego, then the critical question psycholytic therapy concepts need to answer is: How does a singular or a series of acute experiences of psychedelic-induced ego alteration need to be structured so that the ego is optimally situated to recognize and lastingly overcome maldaptive aspects of long-established modes of thinking, emotion regulation, defense, self-representation, and/or relating to others? if we understand the character as the psychological backbone structure that carries the chronic and habitual modes of the ego (see Section 2.2.3), then we must infer that more than the ego itself it is the character and its roots we need to psycholytically reach if we want the ego to accommodate any lasting change. As to this, knowing a person’s character including their preferred set of defenses should facilitate unmasking those aspects of ego armor that are hardened and rigid and may therefore be a core target of psycholysis and other forms of psychedelic-assisted psychotherapy.

The character typologies known to psychology are plentiful (Lenzenweger and Clarkin, 2005; McAdams, 2008) and some of them have also found their way into psychedelic research (e.g., Bouso et al., 2018). From the typologies available, we suggest, the typology recognized by all three diagnostic systems (ICD10, DSM5, and PDM2) appears to be particularly well-suited for alignment with the above discussed model of psycholytic healing. According to the PDM2, there are 12 character types which are thought to exist on a continuum from healthy and adaptive to pathological (e.g., Oldham and Morris, 2012). They are well-described across psychological schools regarding core beliefs (Beck et al., 2015), behavioral drivers (Joines and Stewart, 2002), central conflicts, and preferred sets of defenses (McWilliams, 2011; Lingiardi and McWilliams, 2017). Importantly, there is an empirical foundation relating the individual character types (and their unbalanced use of defenses) to specific emotional themes and recurring motifs of frustrating or traumatic interactions with primary caretakers, and potential points of developmental arrest (Glickauf-Hughes and Wells, 2006). Going with Balint (see Section 2.3.1), it is these points of arrest (i.e., frustrating life events at the core of a person’s defensive apparatus) therapeutic ego regression seeks to recapitulate. Hence, if a therapist knows a client’s character structure, he or she might already know the emotional themes that may emerge with the client’s COEX or tdyst system.

The working alliance between client and therapist is a major predictor of the success of diverse forms of psychotherapy (Horvath and Symonds, 1991; Martin et al., 2000). In line with the significance of *set and setting* (Johnson et al., 2008; Carhart-Harris et al., 2018), this has also been demonstrated for psychedelic-assisted psychotherapy (Murphy et al., 2022). At the core of every therapeutic regression to the roots of a client’s character, there is a need for a *corrective interpersonal experience* strong enough for the client to disband the emotional ties to the frustrating early-life interactions, and specific to the client’s character (Glickauf-Hughes and Wells, 2006). Along the psycholytic model, therefore, a lasting change to the adaptive patterns of the ego may only be achieved if the psychedelic experience succeeds to permeate and rearrange the characterological core of the ego’s habits. Within the psycholytic experience itself, this process is achieved along emotional abreaction and the gain of insight described by Leuner and Grof, and may be facilitated by *symbolic realization* through the therapist (pers. comm. with Torsten Passie, Spring 2023) (Sèchehaye, 1951). Before and after the psycholytic experience(s), respectively, the ego may need to be strengthened as to observing and integrative functions (Blanck and Blanck, 1974; Busch, 1995), and helped in recognition and restructuring of its defense system (Vaillant, 1997). Aim is to establish a more flexible character structure that provides the ego’s habitual modes with a higher degree of freedom, which is well in line with other psychotherapeutic strategies that aim for increased flexibility of behavior and cognition –such as such for instance third wave forms of cognitive behavioral therapy (e.g., Davis et al., 2020; Hayes et al., 2020; Watts and Luoma, 2020).

### 5.3. Limitations and prospects

In our work, we review ego related concepts and provide a theoretical framework meant to psychologically inform on the psychedelic experience and its therapeutic implications. Our review reflects and synergizes ideas of major psychodynamic thinkers, from Freud to Leuner, who for the most part of their clinical research have not followed the quantitative, statistically corroborated paradigm. The constructs discussed are psychological abstractions that across schools of psychology and philosophy may have different connotations. Our review is not meant to set an *absolute* but rather provide a perspective, which we believe could be of value to the understanding of the psychedelic experience. There is a variety of instruments for assessment of ego functioning (Barron, 1953; Bellak and Goldsmith, 1984), character types (Joines, 1992; Davidson, 2008; Oldham and Morris, 2012), basic conflicts (Simmonds et al., 2015), defense mechanisms (Cramer, 2006), emotional breakthrough (Roseman et al., 2019), and client-therapist alliance (Gukasyan and Nayak, 2022), whose implementation into psychedelic research should be worthwhile. Also, cross-disciplinary research on neuroplasticity, epigenetic modifications, and/or neuronal synchronization patterns that accompany psycholytic and other forms of psychedelic-assisted therapy could be promising (e.g., Mertens and Preller, 2021).

Our review focusses on regression to the characterological roots of maldaptive ego patterns as a central element of psychedelic-assisted, particularly psycholytic healing. Importantly, not all psychopathology is equally accessible through regression (Leiper and Maltby, 2004; Maroda, 2012). Clients with weak ego structures or suicidal tendencies, for instance, are contraindications (Madsen and Hoffart, 1996; Passie, 2021). For other indications, the degree of psychedelic regression may need to be adjusted via dose titration and/or spreading over a series of psychotherapeutic sessions. As psychedelics weaken the defenses and the self-other boundary of the ego, and facilitate processes of therapeutic transference, clients become particularly susceptible to suggestions and intrusions from their social environment (Carhart-Harris et al., 2015; Dupuis, 2021). Hence, psychedelic-assisted psychotherapy comes with a strong imperative for a high personal integrity and a professionally dense psychotherapeutic training and education on the part of the therapist (e.g., Phelps, 2019; Brennan et al., 2021; Spriggs et al., 2023).

In line with this review’s theoretical framework, character pathology has been shown to respond particularly well to psycholytic healing (Leuner, 1994). However, we believe that a psychedelic therapy-assisted restructuring or maturation of the character that provides the ego with more flexibility could be beneficial to various other indications as well (e.g., Erritzoe et al., 2018). Disorders that are fed by habitual and repetitive ego maladaptation –for instance, depressive rumination (Barba et al., 2022), addiction and compulsion (Meinhardt and Sommer, 2023; Moreton et al., 2023; Urban et al., 2023), or modes of distorted self-representation like in phantom limb pain, body dysmorphic or eating disorders (Ramachandran et al., 2018; Ledwos et al., 2023)– may particularly well benefit. Lastly, despite the focus on the psycholytic model of therapy in our review, we do not assume that therapeutic concepts of different psychological schools are mutually exclusive. There are various other psychotherapeutic concepts that may complement the psycholytic approach (e.g., Walsh and Thiessen, 2018; Passie, 2021; Yaden et al., 2022). Psycholytic abreaction and assimilation of a disarmed affectivity into a more flexible character structure, for instance, may well be facilitated by cognitive behavioral strategies of *acceptance* and *learning to let go* (Wolff et al., 2020). Overall, our review speaks in favor of a neat delineation of central constructs like ego, self, and character –both on a conceptual and operational basis– as well as for less rigid boundaries between different forms of (psychedelic-assisted) psychotherapy.

## Author contributions

All authors listed have made a substantial, direct, and intellectual contribution to the work and approved it for publication.

## Footnotes

^1^It should be noted that psychodynamic theories have greatly developed ever since the introduction of the drive and ego psychology. Various other lines of psychodynamic thinking exist (e.g., Kohut, 1977; Pereira and Scharff, 2002; Fonagy et al., 2010), and interdisciplinary construct validation remains an important field of modern psychodynamic research (Boeker et al., 2018; Northoff, 2023a). Nonetheless we chose this rather conservative starting point to provide the reader with a basic understanding of the historical foundations and further developments of the ego-construct.

^2^*Cathexis* is a Greek-derived neologism coined *by* James Strachey’s translation of the Freudian term *Besetzung* – a German noun translating as *occupation* or *the act of occupying/of taking possession*. *Cathexis* has widely been adopted by early psychodynamic writers, with varying connotations. In Freud’s own English words, the adjective form of *Besetzung* (i.e., *besetzt*) translates as being *emotionally colored* or as having an *emotive tone* (Ornston, 2002). In our review we understand cathexi*s* as a process, through which the ego makes an object (or its representation) purpose or target of experienced affect excitation (thereby *binding* or attributing the affect to the object). With objects like the own body, cathexis additionally implies claiming ownership and/or embodiment of the object –local anesthesia, for example, artificially deprives body parts of cathexis (thus inducing local *decathexis*) (e.g., Federn, 1952).

^3^Character, temperament, and a person’s value system are the major pillars to what modern psychology understands as *personality*. Historically, however, the terms *character* and *personality* have often been interchangeably used. In line with the PDM2, this review therefore includes references to both *character* and *personality* when discussing character.
